# Quantification of ammonia oxidation rates and the distribution of ammonia-oxidizing *Archaea* and *Bacteria* in marine sediment depth profiles from Catalina Island, California

**DOI:** 10.3389/fmicb.2012.00263

**Published:** 2012-07-24

**Authors:** J. M. Beman, Victoria J. Bertics, Thomas Braunschweiler, Jesse M. Wilson

**Affiliations:** ^1^ School of Natural Sciences, University of California, Merced,Merced, CA, USA; ^2^ Department of Biological Sciences and Wrigley Institute for Environmental Studies, University of Southern California,Los Angeles, CA, USA; ^3^ Department of Marine Biogeochemistry, Helmholtz Centre for Ocean Research Kiel,Kiel, Germany

**Keywords:** nitrification, *amoA*, sediments, bioturbation, *archaea*

## Abstract

Microbial communities present in marine sediments play a central role in nitrogen biogeochemistry at local to global scales. Along the oxidation–reduction gradients present in sediment profiles, multiple nitrogen cycling processes (such as nitrification, denitrification, nitrogen fixation, and anaerobic ammonium oxidation) are active and actively coupled to one another – yet the microbial communities responsible for these transformations and the rates at which they occur are still poorly understood. We report pore water geochemical (O_2_, $NH_{4}^{+}$, and $NO_{3}^{-}$) profiles, quantitative profiles of archaeal and bacterial *amoA* genes, and ammonia oxidation rate measurements, from bioturbated marine sediments of Catalina Island, California. Across triplicate sediment cores collected offshore at Bird Rock (BR) and within Catalina Harbor (CH), oxygen penetration (0.24–0.5 cm depth) and the abundance of *amoA* genes (up to 9.30 × 10^7^ genes g^–^^1^) varied with depth and between cores. Bacterial *amoA* genes were consistently present at depths of up to 10 cm, and archaeal *amoA* was readily detected in BR cores, and CH cores from 2008, but not 2007. Although detection of DNA is not necessarily indicative of active growth and metabolism, ammonia oxidation rate measurements made in 2008 (using isotope tracer) demonstrated the production of oxidized nitrogen at depths where *amoA* was present. Rates varied with depth and between cores, but indicate that active ammonia oxidation occurs at up to 10 cm depth in bioturbated CH sediments, where it may be carried out by either or both ammonia-oxidizing *archaea* and *bacteria*.

## INTRODUCTION

Marine sediments are Earth’s largest microbial habitat, harboring an estimated 10^31^ microbial cells with a total biomass rivaling that of all plants (Whitman et al., 1998). Sedimentary microbial communities play a substantial role in global biogeochemical cycles of carbon (C), nitrogen (N), and sulfur (S) – nearly 50% of N removal from the ocean, for instance, occurs in sediments (Codispoti et al., 2001; Deutsch et al., 2011). Coastal sediments are particularly significant sites for N cycling due to human influence on the global N cycle: agricultural fertilizer use and fossil fuel combustion have more than doubled the amount of N flowing through terrestrial ecosystems, yet over 50% of this N is removed in aquatic and coastal ecosystems before it reaches the sea (Seitzinger et al., 2006; Gruber and Galloway, 2008). The overall size of the N sink in sediments (where N is converted by anaerobic microbial processes into gaseous forms that may flux out of the system) is nonetheless poorly constrained, leading to debate about whether the oceanic N cycle is currently in balance (e.g., Codispoti et al., 2001; Gruber and Galloway, 2008; Deutsch et al., 2011). In order for these outputs to occur via denitrification–which is thought to dominate N loss in sediments at water depths <100 m (Kuypers et al., 2006; Francis et al., 2007) – N must be present in oxidized forms such as nitrite $\left(NO_{2}^{-}\right)$ or nitrate $\left(NO_{3}^{-}\right)$. This is also the case for N loss via anaerobic ammonium oxidation (anammox), as anammox uses $NO_{2}^{-}$ as an electron acceptor (Strous et al., 2006). Dissolved ammonium $\left(NH_{4}^{+}\right)$ must therefore first be oxidized, or reduced N present within organic material must be regenerated and subsequently oxidized, before N can be removed anaerobically.

The oxidation of reduced N occurs via the two-step process of nitrification: ammonia-oxidizing archaea (AOA) and bacteria (AOB) oxidize reduced $NH_{3}/NH_{4}^{+}$ to $NO_{2}^{-}$, and nitrite-oxidizing bacteria (NOB) oxidize nitrite to $NO_{3}^{-}$ (Francis et al., 2007; Erguder et al., 2009). Given the importance of nitrification to sedimentary and global N cycling, AOA and AOB have been studied extensively in estuarine and coastal sediments (Freitag and Prosser, 2003; Mortimer et al., 2004; Bernhard et al., 2005; Francis et al., 2005; Beman and Francis, 2006; Bernhard et al., 2007; Mosier and Francis, 2008; Abell et al., 2010; Wankel et al., 2011) using 16S rRNA or the ammonia monooxygenase subunit A gene (*amoA*) as molecular markers. Most of these studies have targeted surface sediments, and few have examined variability in nitrifier distributions and activity with depth. Surprisingly, Freitag and Prosser (2003) and Mortimer et al. (2004) detected AOB 16S rRNA at depths of up to 40 cm in sediments from Loch Duich in Scotland; based on this observation and detectable rates of nitrification down to 8 cm depth, Mortimer et al. (2004) argue that this is evidence of “anoxic nitrification,” possibly coupled to manganese reduction. Dollhopf et al. (2005) also showed that sediment bioturbation supplies oxygen to AOB present at 6 cm depth in salt marsh sediments.

In contrast to AOB, however, the depth distribution of the recently discovered AOA in sediments is largely unknown. Sulfide inhibits sedimentary nitrification (Joye and Hollibaugh, 1995), but Erguder et al. (2009) argue that AOA tolerate higher concentrations of sulfide than AOB based in part on their presence in sulfidic sediments (Caffrey et al., 2007). In an underground coastal aquifer, Santoro et al. (2008) found that AOA and AOB appear to shift in relative dominance based on salinity and ammonium concentrations (Santoro et al., 2008). Based on pyrosequencing of 16S rRNA, AOA comprised 35% of archaeal sequences in an oxic coral reef sediment sample, but formed a smaller proportion (<10%) of the archaeal community in an anoxic sample (Gaidos et al., 2011). Few other data are available from sediments. Quantifying the distribution of AOA relative to AOB and in relation to nitrification rates may therefore enhance our understanding of sedimentary N biogeochemistry, as no study has collected sediment depth profiles of AOA, AOB, and ammonia oxidation rates in parallel.

**FIGURE 1:**
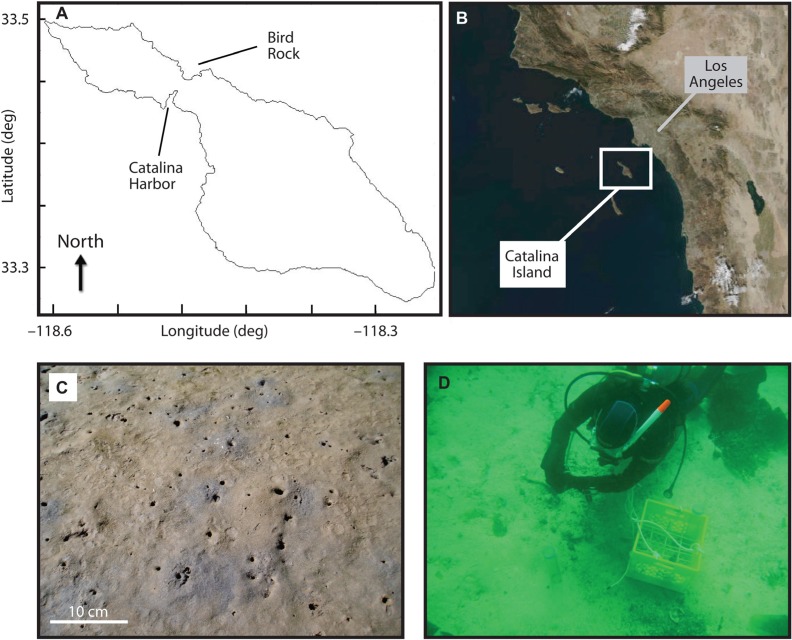
Location of Catalina Island along the coast of California **(B)**, and of Catalina Harbor and Bird Rock sampling locations **(A)**. Catalina Harbor sediment sampling location is shown in **(C)** with scale bar at lower left, and collection of Bird Rock sediments is shown in **(D)**. Burrow density at Catalina Harbor in 2008 was ~120 burrow opening per square meter.

The purpose of this study was consequently to quantify AOA, AOB, and ammonia oxidation rates in sediment cores from Catalina Island, California, USA (**Figure 1**). In a previous study of Catalina Island sediments, Bertics and Ziebis (2009) detected increases in pore water nitrate where decreases in pore water ammonium concentrations were also observed; canonical correspondence analysis revealed that changes in the microbial community with sediment depth were correlated to changes in ammonium concentrations – indicating that ammonium is a key factor influencing microbial communities in Catalina Island sediments. In the present study, AOA and AOB *amoA *genes were quantified in sectioned, triplicate cores collected at two locations, and cores were collected during two sampling periods at one of these locations. Coupled biogeochemical measurements included microsensor oxygen profiles, measurements of dissolved nitrogen in pore waters, and nitrification rate measurements using ^15^N isotopically labeled ammonium. Measurable rates of nitrification were found throughout two cores, and both AOA and AOB *amoA* genes were present at depths of up to 10 cm.

## MATERIALS AND METHODS

### SITE DESCRIPTION

Samples were collected from two locations on or near Catalina Island, California, USA. The first site, “Catalina Harbor” (CH; 33° 27.080′N, 118° 29.293′W), was a shallow (<2 m) intertidal lagoon in CH on the western side of the island (**Figure 1**). The lagoon was a low energy, highly bioturbated area consisting of muddy sand with the majority of grains being <500 μm (Bertics et al., 2010). The two most abundant burrowing macrofauna were the bay ghost shrimp *Neotrypaea californiensis*, Dana, 1854 (Crustacea: Decapoda: Thalassinidea) and the Mexican fiddler crab *Uca crenulata*, Lockington, 1877 (Crustacea: Decapoda: Ocypodoidea). *N. californiensis* inhabits intertidal areas stretching from Alaska to Baja California, and is known to build complex branching burrows that extend to ~76 cm depth and have several openings to the surface (MacGinitie, 1934; Brenchley, 1981; Swinbanks and Murray, 1981). *U. crenulata* is found from Santa Barbara, California to Central Mexico and typically maintains simple J-shaped burrows with a single entrance and that extend to a depth of ~20 cm; *U. crenulata* frequently leaves these burrows during low tide to forage on algae, bacteria, and detritus on the sediment surface (Zeil et al., 2006).

The second site, “Bird Rock” (BR; 33° 25.788′N, 118° 30.314′W), was located 1.5 km off the eastern shore the island in ca. 20 m of water. This site consisted of regions with boulders lying on top of more permeable sandy and gravel sediment (Nelson and Vance, 1979), and regions of rocky outcrops – the largest of which extends out of the water and forms a small island named BR. The sandy region where sampling occurred supported dense patches of the giant kelp *Macrocystis pyrifera* and other brown algae, along with associated meio- and macrofaunal communities. Typical water velocities in the area range from 1 to 7 m s^–^^1^ and the swell surrounding BR ranges from 1 to 3 m in height (Morrow and Carpenter, 2008), making this site an area of high tidal activity in contrast to CH.

### SAMPLE COLLECTION

In 2007, sediment samples from CH were collected on 19 November during high tide, as a minimum of 10 cm of water above the sediment was required to allow for coring, while samples from BR were collected on 21 November below the sea surface via SCUBA in an area near a large rock formation. At both sites, sediment samples were collected using 5 cm diameter, 39 cm length acrylic cores; three intact sediment cores of 5–25 cm sediment depth were collected at each site, and cores were placed in an ice chest at ambient temperature for transport back to the laboratory. In 2008, six sediment cores were collected in approximately the same location in CH as was sampled in 2007, with three parallel cores collected for ^15^N measurements on 14 April, and three parallel cores collected for nutrient measurement, oxygen measurements, and DNA sampling on 15 April.

Following oxygen analyses (see below), each of the nine cores was sub-sampled for ammonium and nitrate concentration analyses and DNA extraction. One-centimeter slices were taken from each core starting at the surface down to 10 cm for the CH cores (CH1–CH6) and 5 cm for the BR cores (BR1–BR3). BR cores extended to a depth of only 5 cm owing to the difficulty in obtaining longer cores from porous sediments via SCUBA. Pore water was collected from each 1-cm slice by centrifugation (10 min at 5000 × *g*) using 50 ml Macrosep^®^ Centrifugal Devices (Pall Corporation, Life Sciences) flushed with nitrogen gas. The recovered pore water (~3 ml) was immediately frozen at -20°C for later determination of dissolved nitrogen compounds.

### PORE WATER AMMONIUM AND NITRATE ANALYSES AND MICROSENSOR OXYGEN PROFILES

Pore water ammonium concentrations were determined by flow injection analysis modified for small sample volumes (Hall and Aller, 1992); 50 μl of pore water was injected for each sediment slice in triplicate. The sum of nitrate and nitrite was determined spectrophotometrically after reduction of samples with spongy cadmium (Jones, 1984). One milliliter of pore water from the respective core slices was used for the colorimetric analysis of nitrite concentrations, and nitrite + nitrate concentrations(after reduction) on a spectrophotometer (Strickland and Parsons, 1972).

Each of the nine intact cores was analyzed for oxygen content on the vertical axis using a Unisense oxygen microsensor – a miniaturized amperometric sensor with a guard electrode (Revsbech and Jørgensen, 1986; Unisense^©^ 2007). For each core, three high-resolution microprofiles of oxygen were measured in vertical intervals of 200–250 μm using Clark-type amperometric oxygen sensors (Revsbech and Jørgensen, 1986; Revsbech, 1989; Unisense^©^, Aarhus, Denmark) following a two-point calibration. Sensors were attached to computer-controlled motorized micromanipulators (Märzhäuser, Wetzlar, Germany) and driven vertically into the sediment in micrometer steps. Signals were amplified and transformed to millivolt (mV) by a two-channel picoammeter (PA 2000; Unisense^©^) and directly recorded on a computer using the software Profix^®^ (Unisense^©^).

### DNA EXTRACTION AND QUANTIFICATION AND QUANTITATIVE PCR ANALYSES

For DNA extraction, ca. 500 mg of sediment from each 1 cm depth interval was stored at -80°C, and DNA was extracted from 200 to 700 mg of sediment using the ZR Soil Microbe DNA Kit (Zymo Research, Irvine, CA, USA; 2007 samples) or the MP Biomedicals FastDNA Spin Kit for Soil (MP Biomedicals, Solon, OH, USA; 2008 samples). DNA was quantified using the PicoGreen assay and the manufacturer’s protocol (Life Technologies Corporation, Carlsbad, CA, USA).

Quantitative PCR (qPCR) analyses were identical to those used by Beman et al. (2012). Archaeal *amoA* qPCR assays used the following reaction chemistry: 12.5 μL SYBR Premix F (Epicentre Biotechnologies, Madison, WI, USA), 2 mM MgCl_2_, 0.4 μM of each primer, 1.25 units AmpliTaq polymerase (Life Technologies Corporation, Carlsbad, CA, USA), 40 ng μL^–^^1^ BSA (Life Technologies Corporation, Carlsbad, CA, USA), and 1 ng DNA in a final volume of 25 μL. β-AOB were quantified using the same reaction chemistry but without additional MgCl_2_. Primers (and relevant references for primer sequences), cycling conditions, qPCR standards, standard curve correlation coefficients, and PCR efficiencies are listed in **Table 1**. All qPCR assays were performed on a Stratagene MX3005P qPCR system (Agilent Technologies, La Jolla, CA, USA).

**Table 1:** Primers (and relevant references for primer sequences) cycling conditions used for qPCR, qPCR standards and standard curve correlation coefficients, and qPCR efficiencies.

Assay	Primers (reference)	Cycling conditions	qPCR standard	r^2^	Efficiency (%)
Archaeal *amoA*	Arch-amoAF and Arch-amoAR	95°C (4 min); 30× of 95°C (30 s),	Clone GOC-G-60-9	0.989-0.994	83.1-101
	(Francis et al., 2005)	53°C (45 s), 72°C (60 s with detection	(GenBank accession no.		
		step); dissociation curve	EU340472) dilution series		
Betaproteobact	amoAF and amoA2R	95°C (5 min); 40× of 94°C (45 s),	Clone HB_A_0206_G01	0.973-0.998	85.7-109
erial *amoA*	(Rotthauwe et al., 1997)	56°C (30 s), 72°C (60 s), detection	(GenBank accession no.		
		step at 81°C (7 s); dissociation curve	EU155190) dilution series		

### NH4+15 OXIDATION RATE MEASUREMENTS

Ammonia oxidation rates were measured by injecting 99 atom percent (at%) NH4+15 solution to a concentration of 33 μmolL^–^^1^ through small silicone-sealed holes drilled into the acrylic core cylinder. The accumulation of ^15^N label in the oxidized $NO_{2}^{-}+NO_{3}^{-}$ pool was measured after incubation for ~24 h. The δ^15^N value of N_2_O produced from $NO_{2}^{-}+NO_{3}^{-}$ using the “denitrifier method” (Sigman et al., 2001) was measured using methods described in Popp et al. (1995) and Dore et al. (1998): N_2_O produced from $NO_{2}^{-}+NO_{3}^{-}$ was transferred from the reaction vial, cryofocused, separated from other gases using a 0.32 mm i.d. × 25 m PoraPLOT-Q capillary column at room temperature, and introduced into ion source MAT252 mass spectrometer through a modified GC-C I interface. Isotopic reference materials (USGS-32, NIST-3, and UH NaNO_3_) bracketed every 12–16 samples and δ^15^N values measured on-line were linearly correlated (*r*^2^ = 0.996–0.999) with accepted reference material δ^15^N values.

Initial at% enrichment of the substrate at the beginning of the experiment (NH4+n, see⁢Eq.⁢1) was calculated by isotope mass balance based on $NH_{4}^{+}$ concentrations assuming that the ^15^N activity of unlabeled $NH_{4}^{+}$ was 0.3663 at% ^15^N. Rates of ammonia oxidation (^15^*R*_ox_) were calculated using an equation modified from Ward et al. (1989):

Rox15=(nt−noNOx−) ×[NO3−+NO2−](NH4+n−oNH4+n)×t,(1)

where *n*_t_ is the at% ^15^N in the $NO_{3}^{-}+NO_{2}^{-}$ pool measured at time *t*, $n_{oNO_{x}^{-}}$, is the measured at% ^15^N of unlabeled $NO_{3}^{-}+NO_{2}^{-}$, $n_{oNO_{4}^{+}}$ is the initial at% enrichment of $NH_{4}^{+}$ at the beginning of the experiment, NH4+n is at% ^15^N of $NH_{4}^{+}$ , and $\left[NO_{3}^{-}+NO_{2}^{-}\right]$ is the concentration of the $NO_{x}^{-}$ pool. All statistical analyses were conducted in MATLAB.

## RESULTS

### MICROSENSOR OXYGEN PROFILES AND PORE WATER DISSOLVED NITROGEN CONCENTRATIONS

**FIGURE 2:**
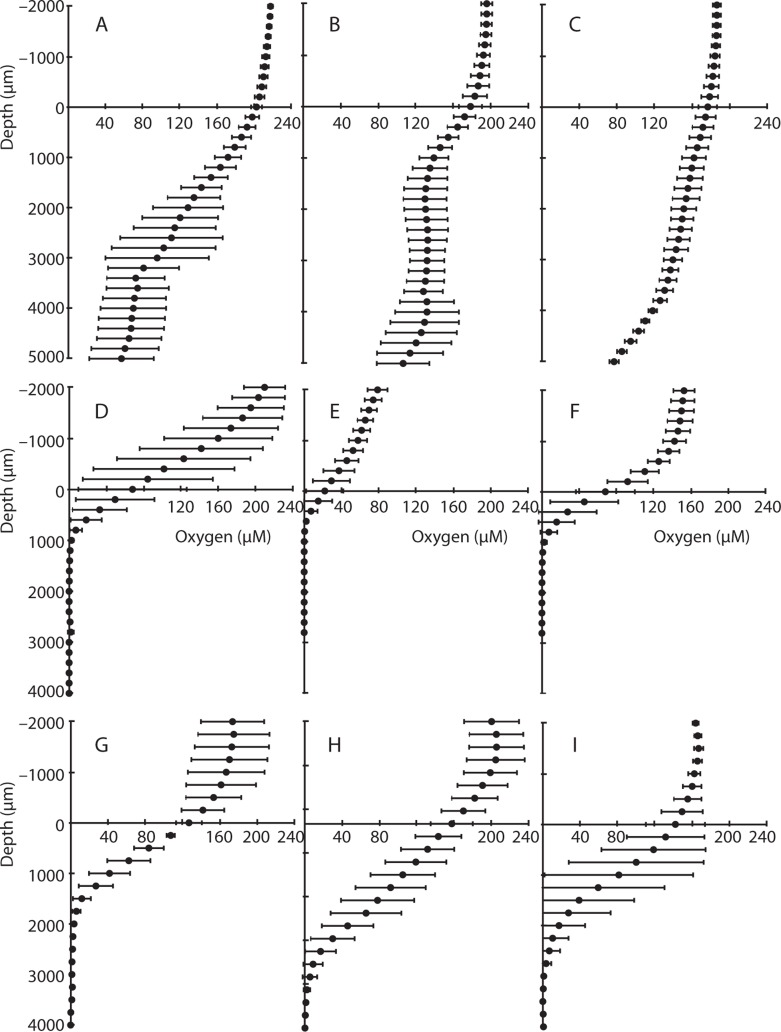
**Microsensor profiles of oxygen in sediment cores**. Data from Bird Rock cores from **(A–C)**, and Catalina Harbor cores from 2007 **(D–F)** and 2008 **(G–I)** are shown; vertical axis depicts depth in sediment (0 μm depth represents the sediment surface and negative values represent overlying water) and the horizontal axis displays oxygen concentrations in micromolar. Error bars denote one standard deviation of triplicate microsensor profiles taken for each core.

Oxygen concentrations in overlying water were similar in both CH and BR sediments in 2007 (typically 150–210 μM), but oxygen concentrations declined to 0 μM at a depth of 2400 μm in CH cores (**Figure 2**), whereas more permeable BR sediments contained >114 μM O_2_ at 2400 μm, and oxygen was detectable down to a depth of 5000 μm (0.5 cm; **Figures 2A–C**). In CH cores collected in 2008, oxygen penetrated up to 3000 μm, consistent with what was observed in 2007. There was substantial variation among measurements made in individual cores, however, and among many of the cores. For example, triplicate measurements in BR core 1 (**Figure 2A**), CH core 1 (**Figure 2D**), and CH core 6 (**Figure 2I**) exhibit high variation, and measured oxygen profiles differed across cores collected at the same time in the same sampling location.

**FIGURE 3:**
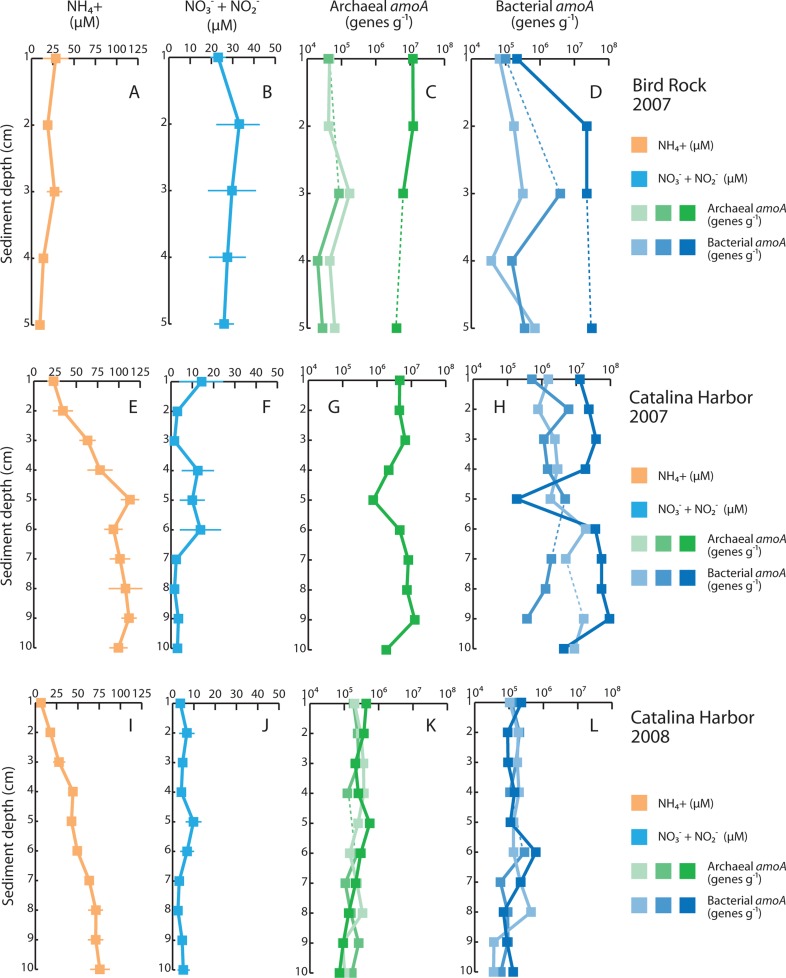
**Sediment profiles of dissolved inorganic nitrogen and ammonia oxidizers**. Average pore water $\left[NH_{4}^{+}\right]$ is shown for Bird Rock in 2007 **(A)**, Catalina Harbor in 2007 **(E)**, and Catalina Harbor in 2008 **(I)**, and pore water $\left[NO_{3}^{-}+NO_{2}^{-}\right]$ is shown for Bird Rock **(B)**, Catalina Harbor in 2007 **(F)**, and Catalina Harbor in 2008 **(J)**. Archaeal *amoA* genes (g-sediment^–^^1^) are shown for individual cores collected at Bird Rock **(C)**, Catalina Harbor in 2007 **(G)**, and Catalina Harbor in 2008 **(K)**. Bacterial *amoA* genes (g-sediment^–^^1^) are shown for individual cores collected at Bird Rock **(D)**, Catalina Harbor in 2007 **(H)**, and Catalina Harbor in 2008 **(L)**. In **(C–L)**, dashed lines denote depths were data values are omitted due to qPCR inhibition of samples, and color shading denotes different cores. Light green/light blue denotes BR1 **(C,D)**, CH1 **(G,H)**, and CH4 **(K,L)**; “mid” green/“mid” blue denotes BR2** (C,D)**, CH2 **(G,H)**, and CH5 **(K,L)**; dark green/dark blue denotes BR3 **(C,D)**, CH3 **(G,H)**, and CH6 **(K,L)**. In **(G)**, archaeal *amoA* was only detectable in one core.

Dissolved nitrogen in pore water also differed between the two sampling locations, but displayed consistent patterns between sampling periods in CH (**Figure 3**). In BR pore water, ammonium $\left(NH_{4}^{+}\right)$ was maximal at 1 cm and declined from 28 to 9.9 μM moving into the sediments. Combined nitrate and nitrite $\left(NO_{3}^{-}+NO_{2}^{-}\right)$ concentrations exhibited moderate variation with depth in BR cores, ranging from 23 to 33 μM. CH sediments differed from BR in absolute values and observed trends of dissolved nitrogen with depth: in 2007, $NH_{4}^{+}$ increased with depth, from 23 to >100 μM; $NO_{3}^{-}+NO_{2}^{-}$ was typically low in CH pore water and reached a maximum value of 14 μM at 1 cm, plateaued at 10–12 μM from 4 to 6 cm, and was below 3.5 μM from 2 to 3 and 7 to 10 cm. The same overall pattern was observed in CH sediments in 2008: $NH_{4}^{+}$ increased from 6.6 to 76 μM with depth whereas $NO_{3}^{-}+NO_{2}^{-}$ concentrations were always less than 10 μM, and exceeded 5 μM only at 2, 5, and 6 cm depth in the cores. On average, concentrations of both $NH_{4}^{+}$ and $NO_{3}^{-}+NO_{2}^{-}$ were lower in 2008 compared with 2007, but these differences were not significant owing to variability between replicate cores. Inter-core variability was generally much higher for $NO_{3}^{-}+NO_{2}^{-}$ than $NH_{4}^{+}$ in both 2007 and 2008: $NO_{3}^{-}+NO_{2}^{-}$ varied from 3.3 to 33 μM at 6 cm depth in 2007, and from 2.6 to 15 μM at 5 cm depth in 2008.

### QUANTIFICATION OF AOA AND AOB

To examine whether ammonia oxidizers were present in these sediments, we extracted DNA and quantified the abundance of AOA and AOB based on *amoA* genes. AOA *amoA* genes, AOB *amoA* genes, or both, were present in all samples from all depths, sampling locations, and time points (**Figure 3**). AOB *amoA* genes were quantified in every sample collected in 2007 at CH and BR, whereas AOA were undetectable in two of three CH cores collected in 2007, and were present at lower abundance in two of three BR cores. Both AOB and AOA *amoA* genes varied with depth in BR and CH cores: AOA *amoA* genes ranged from 4.01 × 10^6^ to 1.22 × 10^7^ genes g^–^^1^ in BR core 3 and 2.03 × 10^4^ to 1.73 × 10^5^ genes g^–^^1^ in cores 1 and 2 (**Figure 3**), while AOB *amoA* genes ranged from 6.55 × 10^4^ to 3.26 × 10^7^ genes g^–^^1^ in the BR cores. AOA and AOB *amoA* genes were highly variable across the replicate cores, however, and this pattern held for CH cores from both 2007 and 2008: for most sediment depths, the coefficient of variation among replicate cores was >1. This is clearly indicative of heterogeneity and patchiness in *amoA* genes in these sediments, and most striking is that fact that AOA *amoA* genes were undetected in two sediment cores collected at CH in 2007, but were detected in the third replicate separated by <50cm. Another possibility is that the *amoA* primers did not successfully amplify the archaeal *amoA* sequence types present in these samples; if so, this indicates that entirely different AOA communities inhabit these cores, and is consistent with heterogeneity and patchiness of *amoA* genes in Catalina sediments.

When AOA *amoA* genes were quantified in the CH3 core collected in 2007, they were correlated with *amoA* genes from AOB (*r*^2^ = 0.936, *P*<0.001) with an AOB:AOA slope of 7.78 (**Figure 3**). It is unlikely that this correlation is an artifact of different DNA extraction efficiencies for different depths, as DNA was extracted from 0.15 to 0.25 g of sediment at each core depth and yielded 316–741 ng of DNA, while both AOA and AOB *amoA* genes varied by more than an order of magnitude. In 2008, AOA and AOB *amoA* genes were more weakly related (*r*^2^ = 0.49–0.55, *P*<0.05) in two of the cores, and uncorrelated in the third (*r*^2^ = 0.03, *P*>0.05). As these relationships indicate, we observed relatively little variability in AOB *amoA*:AOA *amoA* ratios with core depth in BR and CH sediments, yet there were obvious differences between cores, sampling locations, and sampling periods in the relative dominance of AOB and AOA *amoA* genes. With a lone exception, AOB *amoA* was 1.9–46 times more abundant than AOA *amoA* in all BR samples (at 1 cm depth in BR core 3 AOA *amoA* was more numerous), while the ratio of AOB to AOA *amoA* ranged from 0.24 (5 cm depth) to 8.6 (4 cm depth) in the CH3 core collected in 2007. AOA *amoA* was not amplifiable in CH cores 1 and 2 from 2007 and AOB *amoA* was therefore present in substantial greater amounts. In contrast, AOA *amoA* genes were more abundant than AOB in the 2008 CH cores, with AOA *amoA*:AOB *amoA* ratios ranging from 0.86 to 2.9 in CH core 4, 0.77 to 2.9 in CH core 5, and 0.5 to 5.1 in CH core 6.

### δ^15^N AND NITRIFICATION RATE PROFILES

**FIGURE 4:**
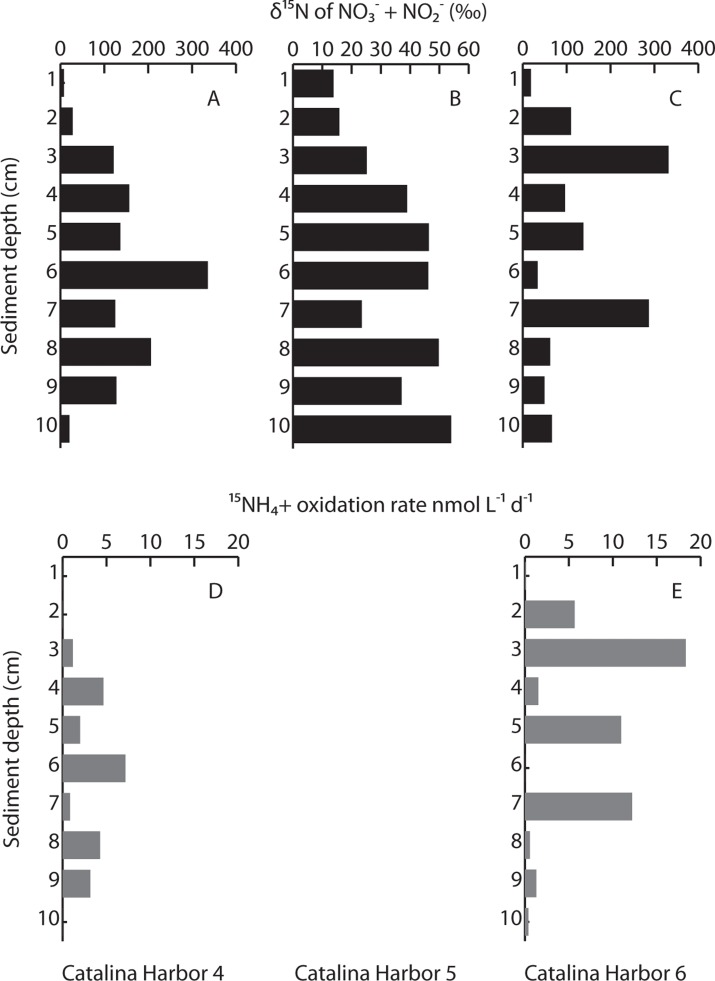
Measured δ^15^N of pore water $NO_{3}^{-}+NO_{2}^{-}$ following incubation with added NH4+15 label **(A–C)**, and NH4+15 oxidation rates **(D,E)** in Catalina Harbor in 2008. Note differences in scales in **(A–C)**; oxidation rates were not calculated in Catalina Harbor core 5 owing to the lack of clear isotopic enrichment.

δ^15^N of $NO_{3}^{-}+NO_{2}^{-}$ in pore water was measured following a 24 h incubation of intact cores collected in 2008 to calculate NH4+15 oxidation rates. δ^15^N of $NO_{3}^{-}+NO_{2}^{-}$ in CH core 5 exhibited only modest enrichment, ranging from 13.8‰ at the surface to 54.0‰ at 10 cm depth (**Figure 4B**). This pattern is typical for sediments (e.g., Lehmann et al., 2007) where denitrification at depth preferentially removes isotopically light N, enriching the remaining $NO_{3}^{-}+NO_{2}^{-}$ pool in ^15^N. Because the values we observed are in the range expected for sedimentary denitrification, this suggests that little or no ammonia oxidation occurred in this core (we enriched the NH4+15 pool to 76.7 at%). Instead, the measured values effectively represent *in situ* δ^15^N of $NO_{3}^{-}+NO_{2}^{-}$, and these values were used to calculate NH4+15 oxidation rates in the other cores. (Two exceptions were the lower δ^15^N values measured at 7 and 9 cm depth, where we instead linearly interpolated the *in situ* δ^15^N values.) In contrast to the δ^15^N values observed in CH core 5, δ^15^N of $NO_{3}^{-}+NO_{2}^{-}$ in pore water exceeded 330‰ in CH cores 4 and 6 (**Figures 4A,C**). Pore water δ^15^N was highly variable throughout each core, and between both cores, and spiked at several depth intervals – indicating that labeled NH4+15 was being oxidized relatively deep within the CH4 and CH6 cores (**Figures 4D,E**). NH4+15 oxidation rate profiles showed maxima at 6 cm in CH4, and at 3 cm in CH6, where rates were also elevated at 5 and 7 cm (**Figure 4**). In both cores, NH4+15 oxidation rates were readily detectable at 9 cm depth. Rates ranged from 0 to 7.15 nmol L^–^^1^ day^–^^1^ in CH4 and 0 to 18.3 nmol L^–^^1^ day^–^^1^ in CH6.

## DISCUSSION

### GEOCHEMISTRY OF CATALINA SEDIMENTS

Oxygen typically penetrates only a few millimeters into coastal sediments owing to rapid consumption during organic matter degradation, or chemical re-oxidation of reduced compounds (Revsbech et al., 1980; Gundersen and Jørgensen, 1990). However, the depth of oxygen penetration can be increased via bioturbation/bioirrigation (Aller, 1982; Ziebis et al., 1996a; Bertics and Ziebis, 2009), sediment permeability and increased bottom water flow velocity, and/or increased wave action (Booij et al., 1991; Precht et al., 2004). Sediment topography features that generate pressure differences can also lead to advective transport of oxygenated water into the sediment (Ziebis et al., 1996b). At BR, sediments contained >114 μM O_2_ at 2400 μm and oxygen was detectable down to a depth of 5000 μm (0.5 cm; **Figures 2A–C**). This is consistent with oxygen transport via advective processes several centimeters into the sediment, especially given the porous nature of these coarse BR sediments. In contrast, in CH sediments, oxygen was not detected below 2400 μm in 2007 (**Figures 2D–F**) and 3000 μm in 2008 (**Figures 2G–I**) – suggesting that oxygen diffuses to a consistent depth at CH. An important caveat to this is the fact that macrofauna can transport oxygen more than 50 cm deep (Ziebis et al., 1996a) and bioturbation has been shown to transfer oxygen multiple centimeters deep into CH sediments (Bertics and Ziebis, 2009). The presence of bioturbation is therefore a likely explanation for the variation within and among many of the CH cores – e.g., CH core 1 (**Figure 2D**) and CH core 6 (**Figure 2I**).

$NO_{3}^{-}+NO_{2}^{-}$ profiles also differed between BR and CH sediments, in that high concentrations (>20 μM) were seen throughout BR cores while concentrations reached a maximum value of 14 μM at 1 cm in CH cores from 2007 and were always less than 10 μM in cores from 2008. However, several subsurface peaks of $NO_{3}^{-}+NO_{2}^{-}$ occurred in CH in both 2007 and 2008, and may reflect either (1) transport of oxidized compounds into the sediment via bioturbation, or (2) production of $NO_{3}^{-}+NO_{2}^{-}$ in the sediment via the activity of nitrifying bacteria and archaea (i.e., *in situ* nitrification). CH cores displayed the typical increase in $NH_{4}^{+}$ that is expected with increasing sediment depth due to microbial remineralization of organic material. Concentrations of both $NH_{4}^{+}$ and $NO_{3}^{-}+NO_{2}^{-}$ were on average lower in 2008 when compared with 2007 – although these differences were not significant owing to variability between replicate cores. A decrease in recruitment of shrimp and a decrease in microbial mat formation was previously observed in these sediments from 2007 to 2008 (Bertics et al., 2010) and may explain this shift in sediment geochemistry. Hence interannual variability in geochemical conditions and microbial activity can occur in CH, but it occurs against a backdrop of substantial spatial variability.

### ABUNDANCE OF AOA AND AOB IN CATALINA SEDIMENTS

Ammonia-oxidizing archaea and AOB were also highly variable in Catalina Island sediments based on the abundance of *amoA* genes. DNA extracted from sediments may not be derived from active or viable microorganisms – indeed, it is possible to recover ancient DNA from sediment cores (Coolen and Overmann, 1998) – yet the presence of, and variability in, oxidized nitrogen at 4–6 cm depth in CH cores is indicative of active production. We assessed this using direct biogeochemical measurements (see below) rather than extraction of RNA, yielding quantitative rates rather than relative levels of gene expression. Our DNA data are nevertheless consistent with other studies profiling AOB in sediments:AOB DNA has been detected at 40 cm depth in Loch Duich sediments (Freitag and Prosser, 2003; Mortimer et al., 2004), 6 cm depth in salt marsh sediments (Dollhopf et al., 2005), and at least 2 cm depth in estuarine sediments from Plum Island Sound (Bernhard et al., 2007), where potential nitrification was measured at up to 4 cm. In these studies, AOB typically ranged from 10^4^ to 10^7^ *amoA* genes g^–^^1^, and our data are similar (3.6 × 10^4^ to 9.3 ×10^7^ *amoA* genes g^–^^1^). However, in addition to AOB, we report *amoA* genes from AOA at up to 5 cm depth in BR sediment cores, and 10 cm depth in CH sediment cores, where they ranged from 7.2 × 10^4^ to 1.3 × 10^7^ genes g^–^^1^.

Previous studies have shown that although AOA and AOB are presumably functionally equivalent, their relative dominance varies across gradients of salinity present in sediments (Caffrey et al., 2007; Mosier and Francis, 2008; Santoro et al., 2008). Studies in soils suggest that pH (Nicol et al., 2008) and $NH_{4}^{+}$ concentrations (reviewed by Erguder et al., 2009) also alter the relative abundance of AOA and AOB – more specifically, an exceptionally high affinity for ammonia benefits AOA when $NH_{4}^{+}$ concentrations are low (Martens-Habbena et al., 2009). While we observed relatively little variability in AOB *amoA*:AOA*amoA* ratios with depth in BR and CH sediments, AOB *amoA *genes were more abundant in BR sediments and CH sediments from 2007, while AOA *amoA* genes were more abundant than AOB in the 2008 CH cores. Different DNA extraction kits were used for CH sediments collected in 2007 and 2008, and it is possible that the MP Biomedicals kit (used in 2008) is less effective in extracting bacterial DNA and so explains the differences observed between the two sampling periods. When comparing measured values, however, 2008 values lie within the range of AOB and AOA *amoA* gene abundances observed across both sites in 2007; this argues against extraction bias, as one would expect much lower or higher numbers for one or both of the genes. In any case, the evidence for interannual variability in ammonia oxidizer populations is mixed, given that: (1) measured $NH_{4}^{+}$ values are still far in excess of *K*_m_ value (123 nM) for the lone cultured marine AOA, *Nitrosopumilus maritimus* (Martens-Habbena et al., 2009), while *K*_m_ values for some AOB are as low as 10 μM (Casciotti et al., 2003 and references therein), and (2) high spatial variation within these sediments might obscure temporal trends. Put another way, our data do not conclusively indicate whether AOA or AOB are more dominant in these sediments, but are indicative of substantial spatial variation and possibly temporal variation as well. This parallels our geochemical results, but there was little correspondence between AOA and AOB and nutrient and rate data: no significant correlations were observed in the 2007 data (all *P*>0.05), whereas AOA were negatively correlated with $NH_{4}^{+}$ – and positively correlated with $NO_{2}^{-}$ – in 2008 (**Table 2**).

**Table 2:** Correlation coefficients (r^2^) for comparisons between qPCR data, nutrient concentrations, and NH4+15 oxidation rates averaged across triplicate cores collected in Catalina Harbor in 2008.

	Log AOA *amoA*	AOA *amoA*	Log AOB *amoA*	AOB *amoA*	$\left[NH_{4}^{+}\right]$	$\left[NO_{2}^{-}\right]$	$\left[NO_{3}^{-}\right]$	NH4+15 oxidation rate
Log AOA *amoA*			0.19	0.07	0.55*	0.44*	0.22	0.28
AOA *amoA*			0.09	0.02	0.48*	0.54*	0.36	0.26
Log AOA *amoA*					0.08	0.03	0.01	0.06
AOB *amoA*					0.02	0.02	0.01	0.02
$NH_{4}^{+}$						0.30	0.03	0.02
NO_2_^-^							0.41*	0.01
NO_3_^-^								0.10

*P < 0.05.

### NITRIFICATION IN CATALINA SEDIMENTS

Ammonia oxidation rate measurements indicated that AOA and AOB were actively nitrifying throughout two of the three collected cores in 2008. Modest enrichment in the CH5 core suggests that although we recovered *amoA* genes, either this DNA was not derived from living organisms, or these organisms were inactive during our incubation. Evidence for the later includes the relatively low δ^15^N values measured at 7 and 9 cm depth, as in a previous study conducted in the same location in 2008, Bertics et al. (2010) found the highest rates of nitrogen fixation at depth of 7 and 9 cm in the most bioturbated location they sampled. Hence one possible explanation for the “light” δ^15^N of $NO_{3}^{-}+NO_{2}^{-}$ at these depths is the oxidation of recently fixed nitrogen, i.e., while ammonia oxidation appeared inactive at the time of our sampling, it may have been previously active within or near these sediment layers. Another explanation for these local minima in the pore water profile is that this represents $NO_{3}^{-}$ and/or $NO_{2}^{-}$ of differing δ^15^N that is present in groundwater.

In the CH4 and CH6 cores, NH4+15 oxidation rates were readily detectable at most depths up 9 cm in both cores, and up to 10 cm depth in the CH6 core. Relatively few ^15^N-based rate measurements have been conducted within sediments (Ward, 2008), but our experimental approach was similar to that used by Mortimer et al. (2004) and our measured rates (0–18.3 nmolL^–^^1^ day^–^^1^) were similar to values of 4.86–89.6 nmolL^–^^1^ day^–^^1^ measured at 2–6 and 10–12 cm depth in Loch Duich (Mortimer et al., 2004). However, our measurements were much lower than the maximum rates measured at 0–2 cm in Loch Duich (1.6 × 10^6^ nmolL^–^^1^ day^–^^1^) and most other measurements in the literature (Ward, 2008). These results therefore capture active NH4+15 oxidation at depths of up to 10 cm in Catalina Island sediments, but also indicate that rates are generally low and variable with depth and between replicate cores.

One possible explanation for measurable ammonia oxidation at depth is the periodic supply of oxygen to aerobic nitrifiers: previous work has shown that alteration of sediment by macrofauna can alter redox chemistry and microbial communities in CH sediments (Bertics and Ziebis, 2009, 2010; Bertics et al., 2010), and burrows were present in the majority of the cores we collected. Previous work by Dollhopf et al. (2005) in fact showed that nitrification rates and AOB abundance were related to burrow abundance. Abiotic “anoxic nitrification” (Mortimer et al., 2004) may also explain oxidation of ammonia at up to 9 cm depth – however, AOB have been detected at greater depths in other sedimentary environments, and *amoA* genes from both AOB and AOA were readily quantified where active ammonia oxidation was also measured. As a result, our findings are consistent with previous work indicating that bioturbation sustains nitrification by providing periodic intrusions of oxygen (Dollhopf et al., 2005; Ward, 2008, and references therein).

Hydrogen sulfide is a confounding issue for nitrification in sediments because it can completely inhibit nitrification (e.g., Joye and Hollibaugh, 1995); yet in spite of relatively high sulfate reduction rates occurring in CH sediments (Bertics and Ziebis, 2010), pore water hydrogen sulfide was not previously detected (Bertics and Ziebis, 2009), possibly because dissolved sulfide reacts with the high levels of iron (Bertics and Ziebis, 2009), leading to the precipitation of iron sulfides (Berner, 1970). Hydrogen sulfide may also be oxidized by sulfide oxidizers present in nearby sediments (Meyers et al., 1987) – in fact, hydrogen sulfide is oxidized by organisms using nitrate as an electron acceptor in oceanic oxygen minimum zones (Canfield et al., 2010). Some combination of these processes likely explains the lack of sulfide inhibition of ammonia oxidation in cores CH4 and CH6.

However, the variation in NH4+15 oxidation rates that we observed (e.g., between cores and with depth) may stem from production of hydrogen sulfide: similar to the rate measurements reported here, sulfate reduction rates are heterogeneous in CH bioturbated sediments, with areas having sulfate reduction rates of 790 nmol $so_{4}^{2-}$ cm^–^^3^ day^–^^1^ separated by only 3–5 cm from areas displaying rates of <5 nmol $so_{4}^{2-}$ cm^–^^3^ day^–^^1^ (Bertics and Ziebis, 2010). It is therefore possible that in some patches of CH sediment, high sulfate reduction rates inhibit nitrification, while in other areas, low sulfate reduction rates allow for the presence of nitrification – thereby explaining the high levels of variation in nitrification rates seen between replicate cores in CH. This hypothesis is supported by Gilbert et al. (1998), in which the authors found that bioturbation led to the close presence of oxic and anoxic microenvironments, which in turn strengthened the proximity and exchanges between nitrification and denitrification in sediments.

Our results are consistent with ammonia oxidation being broadly but patchily distributed in marine sediments, where this key process may be coupled to anaerobic N cycling and loss. The high degree of heterogeneity observed for substrates, products, genes, and biogeochemical activity – laterally, with depth, and through time – demonstrates that sedimentary N cycling is extraordinarily complex. Understanding this complexity and variability will be critical for balancing the N cycle in an era of global change (Gruber and Galloway, 2008; Beman et al., 2011).

## Conflict of Interest Statement

The authors declare that the research was conducted in the absence of any commercial or financial relationships that could be construed as a potential conflict of interest.
